# Sickness absence and disability pension in relation to first childbirth and in nulliparous women according to occupational groups: a cohort study of 492,504 women in Sweden

**DOI:** 10.1186/s12889-020-08730-5

**Published:** 2020-05-14

**Authors:** Charlotte Björkenstam, Krisztina D. László, Cecilia Orellana, Ulrik Lidwall, Petra Lindfors, Margaretha Voss, Pia Svedberg, Kristina Alexanderson

**Affiliations:** 1 Division of Insurance Medicine, Department of Clinical Neuroscience, Karolinska Institutet, Stockholm, Sweden; 2 Department of Global Public Health, Karolinska Institutet, Stockholm, Sweden; 3 Unit of Occupational Medicine, Institute of Environmental Medicine, Karolinska Institutet, Stockholm, Sweden; 4 Department for Analysis and Forecast, Swedish Social Insurance Agency, Stockholm, Sweden; 5 Department of Psychology, Stockholm University, Stockholm, Sweden

**Keywords:** Sick leave, Child birth, Occupation, Disability pension

## Abstract

**Background:**

Childbirth has been suggested to increase sickness absence (SA) and disability pension (DP). This may vary by occupation; however, knowledge in this field remains limited. We explored SA and DP in the years before and after childbirth among women in four occupational groups and those without occupation.

**Methods:**

We studied nulliparous women aged 18–39 years, living in Sweden on December 31, 2004 (*n* = 492,504). Women were categorized into five skill-level based occupational groups and three childbirth groups; no childbirths within 3 years (B0), first childbirth in 2005 with no childbirth within 3 years (B1), and first childbirth in 2005 with at least one more birth within 3 years (B1+). We compared crude and standardized annual mean SA (in spells> 14 days) and DP net days in the 3 years before and 3 years after first childbirth date.

**Results:**

Women in the highest skill level occupations and managers, had less mean SA/DP days during most study years than women in the lowest skill level occupations group. In B1 and B1+, absolute differences in mean SA/DP, particularly in SA, among occupational groups were highest during the year before childbirth. DP was most common in B0, regardless of group and year.

**Conclusions:**

We found that women’s mean SA/DP days before and after first childbirth was higher with decreasing skill-level of the occupational group and these differences were most pronounced in the year before childbirth. DP was most common among women not giving birth, regardless of occupational group.

## Background

Accumulating results suggest that women’s sickness absence (SA) levels increase during pregnancy, both when compared to their levels before and after pregnancy, to their nulliparous counterparts, or to their partner’s [1–10]. Some studies have suggested that women with children have higher SA than their nulliparous counterparts, prompting the load arising from the combination of paid and domestic work (the “double burden”) as a possible explanation [3, 11, 12]. In contrast, other studies – including several of our own – suggest that, though pregnancy is associated with an increase in SA, women who give birth, especially those who give several births, have lower or comparable SA and lower disability pension (DP) levels in periods outside pregnancy than nulliparous women, even after considering demographic, socioeconomic or – through twin designs – genetic and familial environmental factors [6, 7, 9, 10, 13, 14]; these suggest the existence of a health selection into childbirth and/or role enrichment, i.e., having multiple roles such as that of a parent and employee, may have also positive effects on health and SA [12].

It is well-documented in general working populations and in populations of reproductive age that low education, low occupational class, and low income are associated with markedly higher risks of SA [11, 15–18] and that work conditions contribute to the explanation of these associations [19, 20]. A literature review on determinants of SA frequency, found that – besides the individual’s health and lifestyle – sociodemographic factors and the physical and psychosocial characteristics of the workplace are important determinants [21]. Compared to other dimensions of health, SA has been attributed more to the job level (the degree of autonomy and authority at work) within an occupation [22]. A German study observed a clear gradient in SA by job level even after considering other socioeconomic factors [23].

Similarly, women’s high SA during pregnancy may not be explained only by pregnancy-related medical conditions [9]. A Swedish study, investigating the role of work conditions in the relation between pregnancy and SA [24] concluded that levels of maternal SA appeared to be attributed also to non-medical factors, such as; attitudes towards work during pregnancy and latitude of SA criteria, i.e., if these also include common disorders associated with pregnancy that are otherwise not regarded as “illnesses”. High physical and psychological job demands, limited possibilities to adjust the work conditions to the demands of the pregnancy, and positive attitudes towards sick listing may also contribute to pregnant women’s SA [9]. Several of these factors vary by occupation, thus, it is plausible that SA/DP during pregnancy may vary according to occupational group. Similarly, women of high occupational groups may have better coping skills and more autonomy and more flexibility at their workplace, thus better possibilities to meet the challenges arising from combining paid work with pregnancy and with parenthood. A Swedish study analyzed whether the association between being registered to be living with children aged < 18 years and SA differs by education and income among women, but found limited support for such differences [11]. To our knowledge, no previous study has investigated whether the association between childbirth and SA/DP differs by occupational group.

In our recent Swedish study of initially nulliparous women we found that women who gave birth, especially those with several births, had generally lower mean SA/DP days than women who did not give birth and that among parous women mean SA/DP increased mainly in the year before first childbirth and then decreased [9, 10]. In the present study we explored whether SA and DP trends in these three groups of women, i.e., those who remained nulliparous, who gave birth once, and who gave birth more than once during the study period, varied by their type of occupational group.

## Methods

A longitudinal population-based cohort study of initially nulliparous women was conducted. We linked individual-level information from five population-based registers by means of the unique personal identity number, assigned to all residents in Sweden [25].

The Longitudinal Integration Database for Health Insurance and Labor Market Studies (LISA), held by Statistics Sweden, was used to identify women who resided in Sweden on 31 December 2004. We further used this register to obtain information on occupation, country of birth, age, marital status, and year of immigration and emigration, respectively.

The Medical Birth Register (MBR), held by the National Board of Health and Welfare, was used to obtain information on childbirths (including stillbirths). The MBR covers 97–99% of all births in Sweden since 1973 [26] and contains information on the mother’s age, parity, the date of the child’s birth, various maternal characteristics, and pregnancy outcomes. To identify childbirths not included in the MBR, we searched for childbirth-related diagnoses also in the National Patient Register (NPR), also held by the National Board of Health and Welfare; this register was established in 1964 and its coverage became nationwide in 1987 [27]. We obtained information on previous hospitalizations related to childbirth by searching among both primary and secondary diagnoses for the following International Classification of Disease (ICD) codes: ICD-7: 660, 670–678; ICD-8: 650–662; ICD-9: 650, 651, 652, 659X,W/659.W-659.X, 669.E,F,G,H,W,X, and ICD-10: O75.7-O75.9, O80–84. If a childbirth appeared in both registers, we used the information from the MBR.

The Cause of Death Register held by the National Board of Health and Welfare was used to obtain information on date of death of the women included.

Finally, we obtained information from the register Micro Data for Analysis of Social Insurance (MiDAS), held by the Swedish Social Insurance Agency, on SA and DP (start and end dates and extent (full- or part-time)) during the years 2002–2009.

All residents in Sweden aged 16 years and older with income from work or unemployment benefits are entitled to SA benefits from the public sickness insurance system, if unable to work due to disease or injury. SA benefits are paid by the Social Insurance Agency, however, among employed individuals, sick pay is paid by the employer during the first 14 days of a SA spell. In order not to introduce bias regarding those unemployed, we only included SA spells > 14 days. Most have a first qualifying day, and from the eight SA day a sickness certificate from a physician is required. DP can be granted to those aged 19–64 who, due to disease or injury, have permanently or long-term reduced work capacity, even if they have no previous income from work. Both SA and DP can be granted for full-time or part-time (25, 50% or 75%) of ordinary working hours. Approximately 80% of the lost income, up to a certain limit, is covered by SA benefits, while up to 65% of lost income up to a certain limit, is covered by DP.

### Inclusion and exclusion criteria and formation of childbirth groups

The LISA database was used to identify all women aged 18–39 years and living in Sweden on 31 December 2004 and who also had lived in Sweden during the three previous years (2002–2004). Using information on childbirths from 1964 (NPR) (1973 in MBR) through 2009, we created the following three childbirth groups (T_0_ = date of childbirth):

- B1: Women having their first childbirth in 2005, and no childbirths during the three years and 43 weeks after T_0_ (*N* = 14,299);
- B1+: Women having their first childbirth in 2005 and at least one more childbirth during the three years and 43 weeks after T_0_ (*N* = 24,673);
- B0: Women with no childbirth registered neither before nor during the follow-up period (defined as 3 years and 43 weeks from 31 December 2005) (*N* = 453,532).

Thus, women who gave birth to their first child in the 3 years and 43 weeks after 2005 and women who gave birth prior to 2005 were not included in the study. The additional 43 weeks in the above childbirth-group definition was to take into account SA or DP during a possible new pregnancy and to exclude those from the B0 group who were pregnant towards the end of the follow-up. A total of 556 (1.43%) of the 38,972 women in B1 or B1+ were identified only through the NPR. For women who had their first childbirth during 2005, we set the date of delivery as T_0_. For women in B0, T_0_ was set to 2 July 2005. The study period ranged from 3 years prior to 3 years after T_0_.

### Occupation

We obtained information on the women’s occupation in 2004 from LISA. If this information was not available, data on occupation from 2005 was used to increase coverage on occupation. Occupation was coded according to the Swedish Standard Classification of Occupations 1996 (SSYK 1996) [28], which is based on the International Standard Classification of Occupations 1988. We used the first digit in the SSYK codes and categorized occupations into the following four groups and one additional group for the women without occupational information:

- Group 1: Legislators, senior officials, managers, and professionals (SSYK 0–2)
- Group 2: Technicians, associate professionals, and clerks (SSYK 3–4)
- Group 3: Service workers and shop sale workers (SSYK 5)
- Group 4: Skilled agricultural and fishery workers, craft and related trade workers, plant and machine operators, assemblers, and elementary occupations (SSYK 6–9)
- Group 5: No information on occupation.

### Sickness absence and disability pension days

We calculated the number of annual mean net SA and DP days during each of the 3 years preceding T_0_ and the 3 years after for each group (See “Statistical analyses” section). Part-time SA/DP days were combined, e.g., two gross days of half-time SA or DP was counted as one net day.

### Sociodemographic factors

We considered the following variables measured in 2004 from LISA [17]: age (categorized into four groups 18–24, 25–29, 30–34, or 35–39 years), country of birth (four groups: Sweden, other Nordic country, other European Union 25, or rest of the world), type of living area (based on the H-classification scheme [29], categorized as: large city (Stockholm, Gothenburg, Malmö); medium-sized city (≥90.000 inhabitants); or small city/village (< 90,000 inhabitants), and family situation (married/cohabitant or single). Registration on cohabitation is not complete for couples without children, hence there is an underreporting of cohabitations for the years preceding childbirth, that is, in 2004.

### Analyses

We first calculated for all occupational and childbirth groups the proportion of women with any SA/DP during the years studied. Since the great majority of women had no SA/DP days and thus the median in most of these groups was 0 days, we calculated mean SA/DP days in each of our occupational and childbirth groups; thus, though the continuous SA/DP days variable had a skewed distribution, we considered that the mean would be a more sensitive measure of the amount of SA/DP days than the median. We computed crude and standardized annual mean net SA and DP days with 95% confidence intervals (CI), for the three childbirth groups within each of the five occupational groups, starting 3 years before T_0_ and ending 3 years after T_0_. Study years are referred to as e.g., Y_− 3_ (the third year before T_0_), Y_+ 1_ (the year after T_0_). We used a direct standardization using B1 as the standard population. In the standardization, the following sociodemographic variables were taken into account; age, country of birth, type of living area, and family situation. Women who emigrated or died during the study years were excluded the year after emigration or death. All analyses were conducted using SAS 9.4.

## Results

Of the 492,504 women included in this study, most were either in occupational group 3 (31%) or in group 5 (no occupation, 26%) (Table 1). The proportion of the three childbirth groups differed in the various occupational groups. No childbirth (B0) was most common in all five groups, spanning from 81.2% in occupational group 1 to 94.2% in occupational group 4. In group 5, 97.3% had no childbirth (B0). The highest proportion of B1+ was found in occupational group 1 (13.4%) and lowest proportion in group 4 (3.3%) and in group 5 (1.3%). Women in group 5 were in general younger than those in the other groups. Regardless of occupational group, B0 women tended to be younger.

**Table 1:** Characteristics of women by occupational and childbirth groups (*N* = 492,504). Number and percentages

Factors, in December 2004	Group 1: Non-manual occupations ***n*** = 48,442 (10%)			Group 2: Lower non-manual occupations ***n*** = 98,332 (20%)			Group 3: Manual occupations ***n*** = 153,666 (31%)			Group 4: Lower manual occupations ***n*** = 63,971 (13%)			Group 5: Missing ***n*** = 128,093 (26%)		
	B0	B1	B1+	B0	B1	B1+	B0	B1	B1+	B0	B1	B1+	B0	B1	B1+
Total %	81.2	5.4	13.4	87.9	4.0	8.1	93.0	2.8	4.2	94.2	2.5	3.3	97.3	1.4	1.3
Age (years)															
18–24	13.0	2.1	2.0	36.7	9.9	9.9	64.0	35.6	38.7	61.8	35.2	39.6	73.6	62.6	62.9
25–29	33.9	28.2	41.1	29.5	36.9	47.9	20.0	36.3	42.3	17.6	33.6	39.9	11.7	16.2	18.2
30–34	29.8	46.3	48.1	18.6	35.8	35.4	8.9	20.3	16.6	10.1	21.0	17.0	7.7	14.3	14.4
35–39	23.3	23.4	8.8	15.2	17.5	6.9	7.0	7.9	2.4	10.5	10.2	3.5	7.0	6.9	4.5
Country of birth															
Sweden	89.5	91.0	94.4	91.4	91.0	94.3	89.4	86.7	90.6	87.1	83.3	88.9	82.4	73.6	73.7
Other Northern countries	1.7	1.8	1.3	1.3	1.6	1.1	0.8	1.1	0.6	1.0	1.1	0.7	1.1	1.2	0.8
Other EU 25	2.6	1.8	1.2	1.3	1.6	0.9	1.0	0.8	0.7	1.3	2.0	0.9	2.4	1.9	1.8
Rest of the world	6.2	5.4	3.2	6.0	5.7	3.7	8.8	11.3	8.1	10.6	13.6	9.5	14.1	23.3	23.7
Type of living area															
Large cities	55.4	55.9	56.5	51.7	49.4	47.8	41.3	37.1	33.3	35.8	33.6	28.9	40.0	39.4	38.9
Medium-sized cities	30.8	29.4	29.7	32.4	31.9	33.6	36.6	36.4	38.0	39.5	35.0	37.1	36.7	36.5	35.5
Small cities	13.8	14.7	13.8	16.0	18.8	18.6	22.1	26.6	28.7	24.7	31.3	34.0	23.3	24.1	25.6
Family situation															
Married or cohabiting	9.1	34.1	39.0	5.6	25.2	30.0	3.6	16.9	18.2	5.0	15.7	16.4	3.0	19.2	23.8
Single	90.9	65.9	61.0	94.4	74.8	70.0	96.4	83.1	81.8	95.0	84.3	83.6	97.0	80.8	76.2

All characteristics are obtained from LISA 2004. Occupation was obtained from LISA 2004, and if not available, from 2005

*B0* No childbirth; *B1* one childbirth; *B1+* two or more childbirths

The number of SA/DP days/year ranged between 0 and 365/366. Overall, during all study years, SA/DP was most common in occupational group 4 (besides in group 5, where especially DP was high), and was lowest in occupational group 1. Moreover, the absolute majority of the women had no SA or DP, regardless of occupational group or childbirth group (Table 2).

**Table 2:** Number and proportion of women with sickness absence and/or disability pension during the years studied

		Occupation 1			Occupation 2			Occupation 3			Occupation 4			Group 5 missing		
		N	No SA/DP	SA/DP	N	No SA/DP	SA/DP	N	No SA/DP	SA/DP	N	No SA/DP	SA/DP	N	No SA/DP	SA/DP
**Y**_**-3**_	**B0**	39 320	92.1	7.9	86 447	90.6	9.4	142 865	92.0	8.0	60 265	90.3	9.7	124 635	88.3	11.7
	**B1**	2 625	90.8	9.2	3 896	88.1	11.9	4 369	86.2	13.8	1 608	83.1	16.9	1 801	87.6	12.4
	**B1+**	6 497	94.0	6.0	7 989	91.8	8.2	6 432	88.4	11.6	2 098	86.8	13.2	1 657	92.3	7.7
**Y**_**-2**_	**B0**	39 320	92.2	7.8	86 447	91.1	8.9	142 865	92.3	7.7	60 265	90.6	9.4	124 635	87.6	12.4
	**B1**	2 625	90.5	9.5	3 896	87.8	12.2	4 369	86.6	13.4	1 608	84.8	15.2	1 801	85.1	14.9
	**B1+**	6 497	94.0	6.0	7 989	92.3	7.7	6 432	89.3	10.7	2 098	87.1	12.9	1 657	90.4	9.6
**Y**_**-1**_	**B0**	39 320	91.3	8.7	86 447	90.3	9.7	142 865	91.7	8.3	60 265	89.7	10.3	124 635	86.6	13.4
	**B1**	2 625	65.6	34.4	3 896	61.5	38.5	4 369	60.5	39.5	1 608	59.8	40.2	1 801	77.7	22.3
	**B1+**	6 497	74.0	26.0	7 989	68.3	31.7	6 432	64.8	35.2	2 098	64.7	35.3	1 657	80.8	19.2
**Y**_**+1**_	**B0**	39 320	90.6	9,4	86 447	89.8	10.2	142 865	90.8	9.2	60 265	89,0	11.0	124 635	85.4	14.6
	**B1**	2 625	90.8	9.2	3 896	89.3	10.7	4 369	89.7	10.3	1 608	89.6	10.4	1 801	85.5	14.5
	**B1+**	6 497	93.8	6.2	7 989	93.3	6.7	6 432	93.2	6.8	2 098	92.3	7.7	1 657	91.4	8.6
**Y**_**+2**_	**B0**	38 834	90.3	9.7	85 822	89.5	10.5	142 039	90.2	9.8	59 890	88.4	11.6	122 336	84.0	16.0
	**B1**	2 619	92.7	7.3	3 888	90.3	9.7	4 366	88.5	11.5	1 603	88.0	12.0	1 794	85.2	14.8
	**B1+**	6 496	87.1	12.9	7 988	86.1	13.9	6 432	81.9	18.1	2 098	82.7	17.3	1 657	85.6	14.4
**Y**_**+3**_	**B0**	38 251	90.8	9.2	85 103	89.8	10.2	141 106	90.1	9.9	59 457	88.5	11.5	119 403	83.0	17.0
	**B1**	2 590	90.9	9.1	3 866	88.7	11.3	4 347	85.9	14.1	1 597	86.7	13.3	1 783	83.3	16.7
	**B1+**	6 495	83.1	16.9	7 987	81.2	18.8	6 431	78.6	21.4	2 098	79.6	20.4	1 656	81.9	18.1

*SA* Sickness absence, *DP* Disability pension, *B0* No childbirth, *B1* one childbirth, *B1+* two or more childbirths

**Fig. 1:**
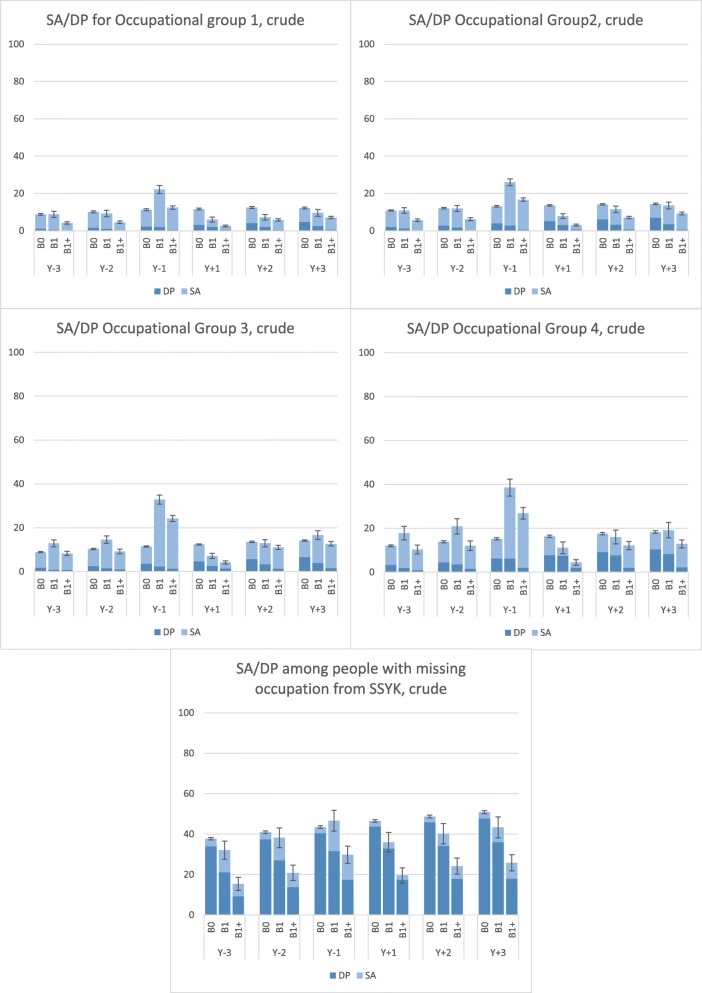
Annual crude mean sickness absence and disability pension days by occupational group, year and childbirth. SA, sickness absence; DP, disability pension; Y_− 3_ to Y_+ 3,_ the 3 years before and 3 years after date of birth/index date; Group 1–5, occupational group; B0, women with no childbirth neither before nor during the follow-up period (defined as 3 years and 43 weeks from 31 December 2005); B1, women having their first childbirth in 2005, and no childbirths during the 3 years and 43 weeks afterwards; B1+, women having their first childbirth in 2005 and at least one more childbirth during the 3 years and 43 weeks afterwards. The vertical lines represent the 95% confidence intervals for the sum of SA and DP net days

When we analyzed crude mean SA days/year, we observed the lowest values in women in B1+ in occupational groups 1 and 2 during all years except at Y_− 1_, and Y_+ 3_, when lowest mean SA days were found among women with no childbirths (B0) (Fig. 1). In occupational groups 3 and 4, women in B0 had the lowest mean SA days (7.9–9.4) during all years except during Y_+ 1_, when women in B1+ had lowest SA net days (2.4 and 2.8, respectively). During the year of pregnancy (Y_− 1_), where the mean SA/DP days/year peaked, we saw a broad range of SA spanning from 20.0 days in occupational group 1 to 32.4 in group 4, among women in B1. DP, on the other hand, was most common in B0, regardless of occupational group and year studied. However, the mean values differed between occupational groups. In group B0 the annual mean DP days/year ranged from 1.2 to 4.8 in occupational group 1, from 2.1 to 7.0 in group 2, from 1.8 to 6.6 in group 3, and from 3.3 to 10.4 in group 4. Highest mean DP days/year was seen in the group 5, ranging from 34.0 to 47.7.

**Fig. 2:**
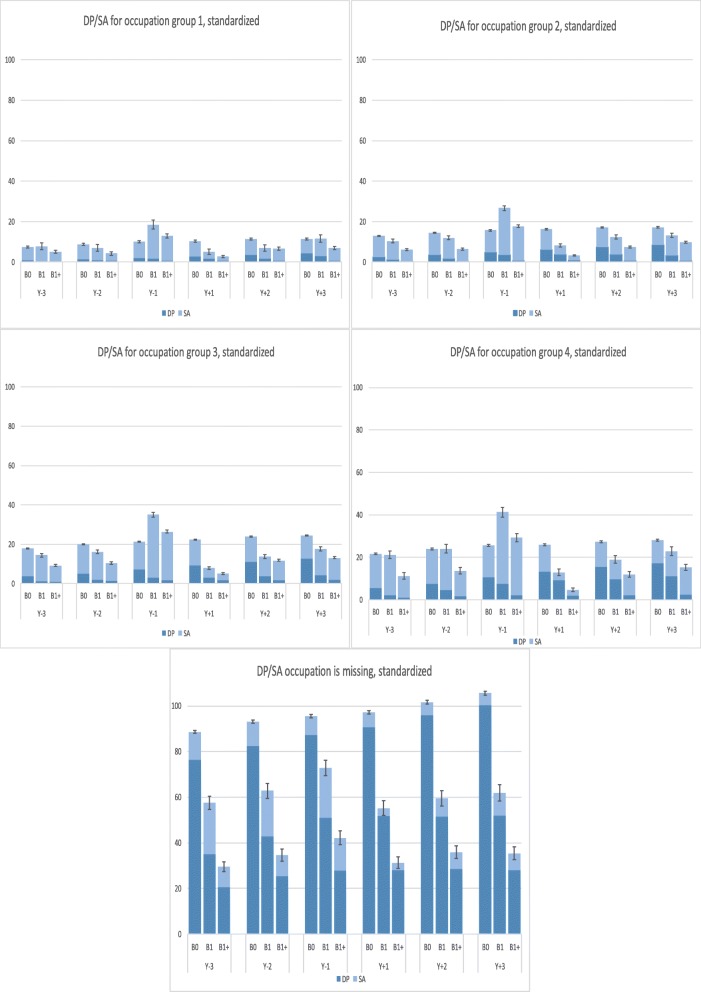
Annual standardized mean sickness absence and disability pension days by occupational group, year and childbirth. SA, sickness absence; DP, disability pension; Y_− 3_ to Y_+ 3,_ the 3 years before and 3 years after date of birth/index date; Group 1–5, occupational group; B0, women with no childbirth neither before nor during the follow-up period (defined as 3 years and 43 weeks from 31 December 2005); B1, women having their first childbirth in 2005, and no childbirths during the 3 years and 43 weeks afterwards; B1+, women having their first childbirth in 2005 and at least one more childbirth during the 3 years and 43 weeks afterwards. The vertical lines represent the 95% confidence intervals for the sum of SA and DP net days

The *standardized* mean SA and DP days/year are displayed in Fig. 2. Also here, women in B1+ had lowest SA in occupation groups 1, 2, and 3 during all years except for Y_− 1_ and Y_+ 2_ (group 1 and 3), Y_− 1_ and Y_+ 3_ (group 2). During those years, women in B0 had lowest number of SA days. In occupation group 4, the B1+ group had lowest SA during Y_− 3_, Y_− 2_ and during Y_+ 1_ while B0 had lowest SA at Y_− 1_. Regardless of occupational group and year, B1+ had lowest mean DP days/year and B0 had highest. However, the mean DP days differed. B0 women in occupational group 1 had 0.9 mean DP days/year, as compared to 0.4 DP days in B1 and 0.5 DP days in B1+ during Y_− 3_. In occupational group 4, women in B0 had 5.6 mean DP days, in B1 they had 2.2, and in B1+ 1.0 mean DP days. The missing group (group 5) stands out regarding DP, during Y_− 3_ the B0-women had 76.5 mean DP days, while those in B1 had 35.0, and those in B1+ had 20.5 mean DP days.

Also, from those figures it is clear that when measuring SA/DP in terms of length, rather than in terms of occurrence (as in Table 2), no group reached the highest possible number per year, i.e., 365 days or was even near that sum.

**Fig. 3:**
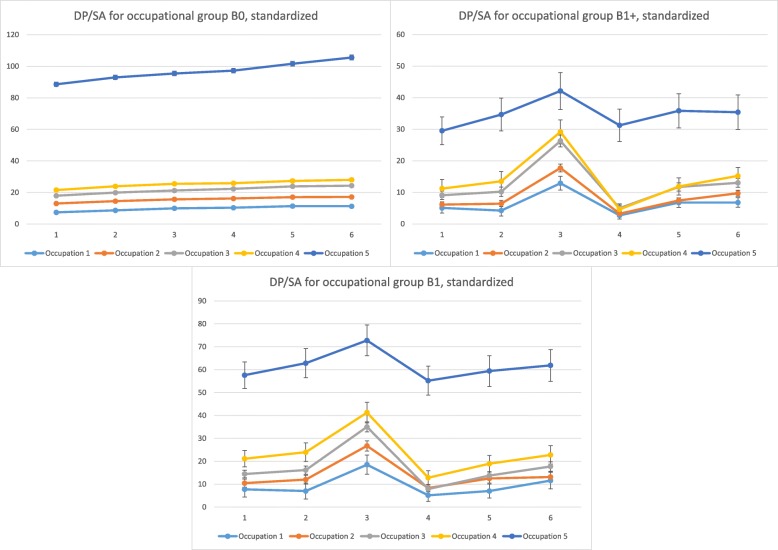
Annual standardized mean sickness absence and disability pension days by childbirth and occupational group. SA, sickness absence; DP, disability pension; Y_− 3_ to Y_+ 3,_ the 3 years before and 3 years after date of birth/index date; Group 1–5, occupational group; B0, women with no childbirth neither before nor during the follow-up period (defined as 3 years and 43 weeks from 31 December 2005); B1, women having their first childbirth in 2005, and no childbirths during the 3 years and 43 weeks afterwards; B1+, women having their first childbirth in 2005 and at least one more childbirth during the 3 years and 43 weeks afterwards. The vertical lines represent the 95% confidence intervals for the sum of SA and DP net days

Figure 3 illustrates the levels of crude mean SA and DP net days/year combined and the corresponding confidence intervals, by the three childbirth and the five occupational groups. Overall, B1+ had fewest mean days. Among women who had at least one childbirth, all occupational groups had a peak at Y_− 1_ followed by a decline.

## Discussion

This large longitudinal cohort study of nulliparous women in Sweden in 2004, revealed that patterns of SA/DP around the years of first childbirth varied according to occupational group, in a graded manner. Beside the group of women with no registered occupation (group 5), manual workers (group 4) had overall the highest mean SA/DP days in the periods immediately before and after pregnancy, and even more so during the time of pregnancy. In contrast, women in higher non-manual occupations (group 1), had the lowest overall mean SA/DP days, including during the year before childbirth. The increase in SA during the year before childbirth was more pronounced with decreasing occupational group. Across most occupational groups, women who had more than one childbirth had the lowest mean SA/DP days, except during the year prior to first childbirth (i.e., when pregnant).

Our findings are in line with a large body of literature that has shown SA/DP to be more prevalent in occupations that require less education [16–18, 30–32] and with the few but increasing number of studies reporting that women who give birth, especially those with more than one birth (up to two or three during our study period), have except during pregnancy less or comparable SA/DP than their counterparts who do not give birth [9, 10, 13, 14], thus suggesting a health selection into giving birth, and especially for having more than one childbirth. In contrast to what has been suggested [3, 11, 12], our findings suggest rather that childbirth is associated with less rather than more SA/DP, apart from during pregnancy.

Our study extends the findings of the previous studies investigating patterns of SA/DP in relation to childbirth by exploring differences across occupational groups in women’s SA/DP in the years shortly before and after first childbirth, including during the period of pregnancy. Our finding that differences in the combined outcome SA/DP – and even more pronouncedly in SA – across occupational groups increased during the year before pregnancy, i.e., when women were pregnant, and were smallest during the year after childbirth, i.e., when most mothers are on parental leave, highlights the possible contribution of the physical and the psychosocial work environment to SA/DP [17]. Prior studies have shown that high physical workload, high job strain, and low job control – work characteristics well-known to differ by occupational class – are associated with higher risks for SA and DP [17, 33–38]. Women in lower occupational groups may e.g., have more limited possibilities to adjust the work environment to the demands of the pregnancy, and may consequently have a higher risk of SA than women in higher occupational groups. Our findings are in line with results of a large Norwegian study, exploring 180,000 employed women, which suggested that the high number of SA days among young first-time pregnant women was due to a preponderance of working class women in this group, who generally have more SA [30].

Our results suggest that the group of women with no occupation have very high levels of combined SA/DP due to many women being on DP throughout the years and thus contributing much to high annual mean SA/DP days. The fact that they to a higher degree were on DP, that is, having such a severe morbidity that it led to long-term or permanent work incapacity, might have affected both their capability to give birth and their decision to not give birth, suggesting a health selection into giving birth. The fact that these women also tended to be younger, might contribute to the fact they may have been too sick early on, to even enter the labor market or to continue their education. However, many of them also had SA, indicating income from work and/or unemployment benefits.

### Strengths and limitations

The strengths of this study include its population-based and longitudinal design, the use of nationwide register data with high completeness and validity and virtually no drop-outs, the very large cohort, and that all women that fulfilled the inclusion criteria were included, not a sample [25, 27]. Also, the use of NPR data in addition to the MBR allowed us to include childbirths not captured by the MBR. Furthermore, we were able to account for factors relating both to childbirth and occurrence of SA/DP such as maternal age, country of birth, family situation, and type of living area, by means of a standardized analyses. Other strengths are that we could use net days instead of gross days, and that all information was based on administrative registers, not self-reports with possible recall bias. It is also an advantage that this study was conducted in a Swedish setting, with a very high employment rate among women – that is, the health selection into the labor market is not as strong as in other countries. However, this study also has some limitations that need to be addressed. First, births among women who had only given birth outside of Sweden would not appear in the registers and these women might, therefore, be incorrectly categorized as B0, that is, as not having had any childbirths. This could lead to differential misclassification of exposure and thus biased levels of SA/DP. To reduce this possible misclassification, and to make sure we had information on their possible SA/DP, we used residence in Sweden in 2002–2004 as an inclusion criteria. Second, the registers used in the analyses only hold data on SA reimbursed by the Swedish Social Insurance Agency; thus SA spells < 15 days were not included in our analyses. Although this means that the number of SA days per year is somewhat underestimated in all groups, it is not likely that the resulting misclassification of SA/DP would differ substantially by childbirth or occupational group. Moreover, the short SA spells account only for a limited number of the total number of SA days during a year [39]. Third, we did not have data on other aspects that might be associated with SA/DP, such as life style, morbidity, pregnancy symptoms, attitudes towards work and being on SA. Fourth, our findings may be generalized only to countries with a high employment rate among women and with a welfare system comparable to that of Sweden in terms of universal provision of SA/DP and high-quality and almost free healthcare.

## Conclusions

In conclusion, we found that trends in women’s SA and DP in the years before and after first childbirth varied by occupational groups, and with having an occupation or not. The less education and skills needed in the occupation, the higher the number of mean SA/DP days in the period before and after childbirth, with this difference being most pronounced during the year before childbirth, i.e., during pregnancy. Mean SA/DP days were highest in the group of women with no occupation before giving birth.

## Data Availability

The sensitive and confidential data used in this study cannot be made public according to the General Data Protection Regulation, the Swedish law SFS 2018:218, the Swedish Data Protection Act, the Swedish Ethical Review Act, and the Public Access to Information and Secrecy Act. Readers may contact Professor Kristina Alexanderson (kristina.alexanderson@ki.se) regarding the data.

## Abbreviations

SA: Sickness absence
DP: Disability pension
B0: Women with no childbirth neither before nor during the follow-up period (defined as 3 years and 43 weeks from 31 December 2005)
B1: Women having their first childbirth in 2005, and no childbirths during the three years and 43 weeks afterwards
B1 +: Women having their first childbirth in 2005 and at least one more childbirth during the three years and 43 weeks afterwards.
LISA: Longitudinal Integration Database for Health Insurance and Labor Market Studies
MBR: Medical Birth Register
NPR: National Patient Register
ICD: International Classification of Diseases
MiDAS: Micro Data for Analysis of Social Insurance
T_0_: Date of first childbirth or 2 July 2005
SSYK: Swedish Standard Classification of Occupations
CI: Confidence intervals
Y_-3_: The third year before T_0_
Y_+1_: The year after T_0_
Y_-2_: The second year before T_0_
Y_-1_: The first year before T_0_
Y_+2_: The second after T_0_
Y_+3_: The third year after T_0_.
